# Bridging clinical and educational contexts: mentalization-based techniques in educational settings

**DOI:** 10.3389/fpsyg.2026.1797274

**Published:** 2026-06-04

**Authors:** Gali Chelouche-Dwek, Oliver Petrick, Brenda McHugh, Neil Dawson, Matthew Hillman, Peter Fonagy

**Affiliations:** 1 Research Department of Clinical, Educational and Health Psychology, University College London, London, United Kingdom; 2 Anna Freud Centre, London, United Kingdom

**Keywords:** classroom behaviour management, coding-scheme, educational settings, emotional regulation, mentalization, mentalization-based interventions, teacher practices, reflective functioning

## Abstract

**Background:**

Mentalization, the capacity to understand one’s own and others’ behaviour in terms of underlying mental states such as intentions, feelings, and beliefs, has well-established clinical applications, including Mentalization-Based Treatment (MBT) and its adapted variants for children and adolescents. Despite growing evidence for its effectiveness in therapeutic settings, little research has examined whether mentalization-based techniques can be systematically applied and evaluated in educational contexts, particularly for children displaying disruptive behaviour and emotional dysregulation.

**Aim:**

This study investigates whether mentalization-based techniques are effective in educational settings and analyses the performance of different techniques in resolving disruptive behaviour and incidents of emotional dysregulation.

**Methods:**

A mixed-methods design was used. An adapted version of an existing framework codifying 13 MBT-derived techniques was applied to teacher–child classroom interactions. Observations were conducted across two classrooms in an alternative provision school in London. In total, 259 distinct incidents were recorded, comprising 815 individual teacher–child interactions. Quantitative analysis used generalised linear mixed models; qualitative analysis drew on theory-driven thematic coding of interaction transcripts.

**Results:**

Analysis revealed that a mentalization approach outperformed non-mentalizing strategies in resolving classroom incidents. Among the specific techniques, *Exploring Mental States in Relationships* and *Exploring Mental States in Others* were most effective, while *Clarification and Exploration* and *Addressing Contradictions* performed no better than non-mentalizing strategies.

**Conclusion:**

The findings add to the emerging evidence for mentalization-based approaches as a means of supporting successful educational environments for both teachers and children. While the results cannot be interpreted as causal, they highlight the promise of incorporating mentalization-based programmes in schools. Future research should employ controlled experiments to further establish the effectiveness of mentalization techniques in addressing disruptive behaviour and emotional dysregulation in the classroom.

## Introduction

### Overview of mentalization

A usually upbeat student is staring out of the window instead of working. The teacher pauses. *Are they bored, daydreaming, or worried about something at home?* Choosing to approach the child, guided by curiosity about what might be happening for them, rather than simply reprimanding them, the teacher draws on the same capacity that underlies all sensitive human relationships: the ability to hold another person’s mind in mind. This is an example of a teacher mentalizing. Mentalization refers to the process of understanding one’s own and others’ mental states such as intentions, beliefs, desires, and feelings (Bateman and Fonagy, 2004; Fonagy et al., 2002). It is a key aspect of successful human functioning (Fonagy and Target, 2006), and strong mentalizing skills are central to developing empathy, regulating emotions, and navigating social interactions effectively (Fonagy and Target, 2002). When this ability is impaired, it can contribute to difficulties such as impulsive behaviour and aggression (Bateman et al., 2023). Whether that momentary act of wondering – and the techniques it generates – can be systematically identified, categorised, and evaluated in educational settings is the central question of the present study.

Fonagy et al. (2002) situate mentalization within early attachment relationships, demonstrating how the capacity to understand mental states develops through sensitive caregiving and shapes the structure of the self. Building on this developmental foundation, the MBT literature identifies four dimensions along which mentalizing varies: the degree to which it is automatic versus controlled; whether the focus is on one’s own mental states or those of others; whether it draws on internal experience or observable external behaviour; and whether the mode is cognitive or affective (Bateman et al., 2023; Fonagy et al., 2002). These dimensions are not independent – optimal mentalizing requires flexibility across all of them – and this multi-dimensional character guided the analysis of teacher techniques in the present study.

### Mentalization in the developmental context

The capacity for mentalization develops across childhood within attachment relationships (Fonagy and Target, 2002). From birth, infants are biologically prepared for social interaction, engaging in reciprocal communication, monitoring caregivers’ responses, and sharing attention (Tomasello and Farrar, 1986; Csibra and Gergely, 2009). At around 18 months, toddlers begin to show self-awareness by recognising themselves in a mirror (Bukatko and Daehler, 2001), which later extends to an understanding of others. By age three, most children can identify basic emotions and recognise that others have distinct feelings and desires (Weimer et al., 2012; Denham et al., 2014). A key milestone is success on false-belief tasks, indicating the ability to represent perspectives that differ from one’s own (Happé and Frith, 2014). Most children pass first-order false-belief tasks at around four years of age (Wimmer and Perner, 1983; Happé and Frith, 2014), while higher-order false-belief understanding, attributing beliefs about beliefs to others, emerges later during middle childhood (Osterhaus et al., 2016).

As children enter school, their social environments broaden and self-concepts become increasingly shaped by abilities and peer comparison (Nelson, 2003; Harter, 2015). This supports the emergence of self-evaluative emotions such as pride and shame (Thompson and Lagattuta, 2006), as well as socially strategic behaviours such as the use of white lies (Weimer et al., 2012). During middle childhood, mentalization becomes more complex and nuanced. With adult scaffolding, children increasingly understand themselves and others in terms of stable internal states, express emotions with greater differentiation, and solve more complex social problems, including higher-order false-belief tasks (Southam-Gerow and Kendall, 2002; Ensink et al., 2015; Osterhaus et al., 2016).

In early adolescence, mentalization develops further in the context of heightened sensitivity to peer evaluation. Adolescents show increased anticipation of social rejection (Gunther Moor et al., 2012), which has been linked to changes in functional connectivity within the brain’s mentalizing network (Burnett and Blakemore, 2009; Sebastian et al., 2011). Pubertal hormonal changes and ongoing prefrontal cortical maturation during this period further modulate the social brain networks that underpin mentalizing (Blakemore, 2012). Overall, mentalization comprises a set of interrelated processes that unfold hierarchically over development rather than a single, unified skill (Malle, 2021). While this account may broadly capture neurotypical development, children who have experienced trauma or who have Special Educational Needs and Disabilities (SEND) may show less advanced mentalizing capacity (Midgley et al., 2017).

### Breakdowns and challenges in mentalizing

While temporary breakdowns in mentalizing are a normal part of life, particularly under stress, they become problematic when they are chronic or context-specific (Midgley et al., 2017). A child’s capacity to mentalize may be underdeveloped for multiple reasons, including genetic factors, as in Autism Spectrum Disorder (ASD) (Frith, 2004), or adverse relational environments such as trauma, foster care, or parental psychiatric difficulties that impair caregivers’ own mentalizing capacity (Ostler et al., 2010). Children with difficult temperaments may therefore require especially attuned and reflective parenting to support the development of mentalization (Aron et al., 2005; Kochanska et al., 2007).

Under conditions of stress or heightened emotion, breakdowns in mentalizing often manifest through specific non-mentalizing modes of thought, as described by Bateman et al. (2023). In teleological mode, internal states are experienced as real only when they lead to observable outcomes (e.g., equating receiving money with being loved). In psychic equivalence mode, internal experience is fused with external reality, so that beliefs (such as being treated unfairly) are experienced as unquestionable facts. In pretend mode, internal experience becomes disconnected from external reality: mental states are spoken about as though they are real, but they are cut off from genuine emotional engagement or meaningful consequence (Bateman et al., 2023). Thinking and feeling become decoupled; a child may speak at length about their feelings or engage in seemingly reflective talk without any genuine processing of inner experience, and the appearance of mentalizing is present while its substance is absent.

Additioanly, a child’s capacity to mentalize is strongly shaped by early attachment relationships and exposure to trauma (Midgley et al., 2017). Insecure attachment takes different forms with distinct effects: anxiously attached children tend to become easily overwhelmed by emotion and remain reliant on others for regulation, whereas avoidantly attached children suppress emotional experience and rely excessively on self-sufficiency, leaving them disconnected from their own internal states (Fonagy et al., 2016). Both patterns, create vulnerabilities for mentalizing breakdown under stress.

Trauma, including abuse and neglect, is particularly damaging (Allen et al., 2012). Research has shown that trauma-specific impairments in reflective functioning are distinct from general mentalizing difficulties and predict relational difficulties above and beyond general reflective functioning (Ensink et al., 2014). In children, maltreatment is associated with a characteristic shift toward hypervigilance – in which attention is directed toward detecting threat rather than understanding others’ intentions – that further undermines mentalizing capacity under stress (Allen et al., 2012). Maltreated children frequently show further mentalizing deficits, including reduced empathy (Klimes-Dougan and Kistner, 1990) and limited engagement in dyadic play (Valentino et al., 2011).

Difficulties in mentalization are implicated across a wide range of childhood psychopathology. In conduct disorders, externalising behaviours have been associated with lower parental mindedness (Meins et al., 2013) and a tendency to attribute hostile intent to others (Dodge et al., 2002). These patterns are closely linked to broader problems of emotional dysregulation (Beauchaine and Cicchetti, 2019), in which intense affect undermines mentalizing and generates cycles of non-mentalizing interaction (Allen and Fonagy, 2006). Importantly, such cycles can be interrupted: interventions targeting mentalizing deficits have been shown to reduce aggression, including in antisocial presentations (Fonagy and Luyten, 2018; Fonagy et al., 2025).

In depression, impairments in mentalization are thought to contribute to negative cognitive biases (Luyten et al., 2012), and children’s capacity to mentalize about themselves and attachment figures is inversely related to depressive symptoms (Ensink et al., 2016). For children with Special Educational Needs and Disabilities, such as ASD, there may be intrinsic limitations in mentalizing, sometimes described as *mind-blindness* (Baron-Cohen et al., 1985). Nevertheless, emotion-based and relational interventions have been shown to improve emotional competence in this group (Ratcliffe et al., 2014).

### Mentalization for adolescents and children

These principles have been adapted for both children and adolescents. For example, MBT for adolescents (MBT-A) targets young people with complex emotional needs, combining structure with relational support (Bevington et al., 2013). MBT for children (MBT-C) was developed by Midgley et al. (2017) as an integrated model for children experiencing difficulties including behavioural problems, low mood, and anxiety. Crucially, MBT-C is grounded in attachment theory and works not only with the individual child but within their wider relational system: parents, caregivers, and other key figures are active participants in the process, with the aim of strengthening the mentalizing environment that surrounds the child (Midgley et al., 2017; Conway, 2024). MBT-C also adopts a play-centred approach, reflecting the understanding that play is a precursor to and vehicle for mentalizing (Fonagy and Target, 1996). The present study asks whether the core mentalizing techniques embedded in this clinical model can be meaningfully identified and evaluated in everyday classroom interactions - a setting that, like the therapeutic context, is defined by sustained relational contact between an adult and a child.

Muñoz Specht et al. (2016) conceptualised Mentalization-Based Interventions (MBIs) used by psychodynamic child and adolescent therapists into a structured framework. Their work was informed by two key texts: *Mentalization-Based Treatment for Adolescents with Borderline Traits* (Fonagy et al., 2014) and *Mentalizing in Clinical Practice* (Allen et al., 2008). Using a combined deductive and inductive approach, they coded transcripts from two psychodynamic therapists’ sessions and identified additional techniques beyond those described in the manuals. The resulting framework was unified by the mentalizing stance principle, emphasising the therapist’s curiosity and respect for the patient’s internal world (Allen et al., 2008). Within this stance, 24 identified techniques were grouped into three categories. The first, supporting mentalizing stance interventions, includes foundational techniques such as empathy and clarification that create the conditions for mentalizing to occur. The second, basic mentalizing techniques, consists of interventions that directly stimulate a patient’s ability to reflect on mental states. The third, specific to work with children, is mentalizing the play context, using play as a medium to heighten awareness of self and others’ minds.

### Mentalization-based approaches in education

In education, a teacher’s capacity to mentalize plays a central role in creating a supportive learning environment that accommodates diverse student needs (Hattie, 2008). Mentalization theory therefore offers a valuable framework for educator training, promoting reflective practice and strengthening student–teacher relationships (Nišponská, 2022; Valle et al., 2016).

The first systematic review of mentalization-based interventions (MBIs) in schools, conducted by Chelouche-Dwek and Fonagy (2025), synthesised evidence from 21 studies out of 5,250 screened articles. Findings indicated that school-based interventions incorporating mentalizing principles demonstrated improvements across a range of socio-emotional outcomes, including empathy, Theory of Mind (ToM), classroom behaviour, and peer relationships (Bianco and Lecce, 2016; Doron, 2016; Finne and Svartdal, 2017; Iuso et al., 2022). ToM training is particularly relevant in middle childhood, supporting both social navigation and academic functioning (Lecce et al., 2014), and embedding such discussions within the curriculum has been shown to facilitate development as children’s social worlds expand beyond the family (Caputi et al., 2021). Strengthening mentalizing capacity also supports self-regulation and more harmonious relationships, particularly when teachers are trained to integrate these principles into everyday classroom practice (Bak et al., 2015; Bateman and Fonagy, 2016; Eppler-Wolff et al., 2020).

A well-established whole-school MBI is the Creating a Peaceful School Learning Environment (CAPSLE) programme, developed in the USA in the early 1990s to reduce aggression and bullying by cultivating a culture of mentalizing (Twemlow et al., 2001; Twemlow et al., 2005). CAPSLE combines a positive climate campaign, classroom management strategies, physical education, and peer and adult mentorship, with a focus on empowering bystanders to restore mentalizing and de-escalate conflict. In a randomised controlled trial, the programme was shown to reduce bullying, classroom disruption, and off-task behaviour compared to controls (Fonagy et al., 2009).

Teacher-focused interventions have also shown promise. Valle et al. (2016) evaluated the Thought in Mind (TiM) Project in Italy, comparing students taught by a teacher trained in mentalizing with those in a control classroom. Only students in the TiM condition demonstrated significant improvements in third-order belief understanding and a shift towards a more adaptive, rational mentalizing style. Although limited by sample size, the study provides preliminary evidence that enhancing teachers’ mentalizing capacity can positively influence students’ mentalizing development.

Mentalization-based approaches in education align with broader initiatives aimed at supporting children’s wellbeing. Growing recognition of the impact of childhood trauma has led to the adoption of trauma-informed teaching practices, which encourage a shift from asking *“What is wrong with you?”* to *“What is happening with you?”* (Phifer and Hull, 2016; Thomas et al., 2019). These approaches are complemented by social–emotional learning initiatives that promote empathy, self-awareness, and decision-making within the curriculum (Durlak et al., 2011; IntegratED, 2023; Kim et al., 2024), as well as programmes supporting teachers’ emotional regulation and compassion (Jennings et al., 2017). Together, these strategies are consistent with current UK Department for Education guidance, which emphasises proactive support for positive behaviour and the use of de-escalation techniques over punitive responses (Department for Education, 2024).

### The present study

As highlighted in the review by Chelouche-Dwek and Fonagy (2025), although a growing number of school-based programmes incorporate mentalizing, the specific techniques are rarely defined, and their methods of application have not yet been systematically observed. Furthermore, no agreed framework exists for identifying, categorising, and evaluating mentalizing techniques in educational contexts.

The present mixed-methods study aims to address this gap. By adapting the therapeutic framework developed by Muñoz Specht et al. (2016) for an educational setting, this research uses naturalistic, audio-based classroom observations to identify the mentalization-based strategies employed by teachers. The efficacy of these techniques is evaluated according to their effectiveness in resolving disruptive behaviour and emotional dysregulation.

This study explores the following research questions:

1. How effective are mentalization techniques used in the classroom when translated from the MBT therapeutic context?
2. How do specific aspects of mentalization perform in the educational context?

Based on the evidence reviewed, three directional hypotheses are proposed. H1: Teacher-led mentalizing techniques will achieve significantly higher rates of incident resolution than non-mentalizing approaches, such that each of the three mentalization categories will outperform the non-mentalizing baseline. H2: Higher-order mentalization techniques (Basic Mentalizing Techniques; Contextualising the Learning Environment) will outperform the foundational Supporting the Stabilising Stance category. H3: Given the documented salience of peer relationships and social context during middle childhood and early adolescence (Crone and Dahl, 2012), techniques explicitly focused on exploring the mental states of others and of relational dynamics (Techniques 5 and 6) will show the strongest effects. This study seeks to provide a foundation for further research, advancing theoretical understanding while supporting the practical development of reflective and responsive learning environments.

## Methods

### Design

This study employs an explanatory sequential mixed-methods design (Creswell and Plano Clark, 2017): quantitative analysis first establishes which techniques are more or less effective, and the subsequent qualitative analysis provides a process-level account of how and why those differences arise, drawing on the same incident data. The two strands are epistemologically integrated rather than sequential add-ons: the qualitative analysis is explicitly guided by the quantitative findings, and convergences and divergences between the two forms of evidence are treated as analytically informative. This design was preferred over a purely quantitative approach because evaluating mentalizing techniques requires understanding of the relational and contextual processes that statistical models alone cannot capture, and over a purely qualitative approach because the scale of the dataset (815 interactions) makes systematic quantitative analysis both feasible and necessary.

The main emphasis is on the quantitative analysis, offering a data-driven assessment of the effectiveness of the techniques used. To complement these findings, a qualitative analysis provides additional depth and context, illustrating how MBT techniques may translate into classroom practice.

### Educational and therapeutic school setting

The study took place in an alternative provision school in London, offering short- and long-term placements outside mainstream education for students with complex emotional, behavioural, and mental health needs. A substantial proportion of students had psychological diagnoses or were in the process of assessment, and many had experienced significant early adversity, including trauma and loss. Staff at the school receive trauma-informed training to support pupils’ emotional regulation and learning.

The school provides an integrated educational and therapeutic provision, combining high-quality teaching methodologies with evidence-based practices drawn from contemporary Child and Adolescent Mental Health Services (CAMHS). Educational and therapeutic input is delivered within a systemic, multi-family-based framework. This describes an approach in which difficulties are understood within the context of family and social systems, and in which interventions engage parents, caregivers, and sometimes groups of families together. It is designed to address unhelpful relational and behavioural patterns and support sustainable change.

Within this broader framework, mentalization-informed practice constitutes one of several guiding approaches, alongside systemic family work and trauma-informed educational strategies. Mentalizing principles are integrated into everyday classroom practice to support staff and pupils in understanding behaviour in terms of underlying mental states, rather than being delivered as a standalone intervention.

#### Participants

Participants were recruited through convenience sampling: the school was selected because it was an established partner institution and because its trauma-informed framework made it an appropriate naturalistic context for the study. The implications of this for generalisability are discussed in the limitations section.

Participants included both students and teaching staff from two classrooms. For this study, the term *teacher* is used inclusively to refer to both lead teachers and teaching assistants, ensuring consistency in describing observed interactions. Owing to small class sizes, staff were often able to work one-to-one with students, including when helping to resolve incidents.

In total, 12 children participated (10 male, 2 female; overall mean age = 9.25 years). All met the school’s criteria for alternative provision, reflecting complex educational, emotional, and/or social needs. Confirmed diagnoses at the time of the study included: Attention-Deficit/Hyperactivity Disorder (ADHD), ASC, Dyslexia, and Oppositional Defiant Disorder (ODD). Several children were undergoing assessment and did not yet have confirmed diagnoses. Many had documented histories of adverse childhood experiences (ACEs; Felitti et al., 1998). The sample was predominantly male (10 male, 2 female). Research documents sex differences in externalising behaviour (Beauchaine and Cicchetti, 2019), emotional regulation, and teacher–student interaction patterns; these differences constitute a potential confounding variable.

In Classroom 1 (5 M, 1F), the children had a mean age of 9.0 years, and the lead teacher had 3.5 years of professional experience. In Classroom 2 (5 M, 1F), the mean age of the children was 9.5 years, and the lead teacher had 9.5 years of professional experience. Across both classrooms, the school maintained a one-to-one student–teacher ratio. The two lead teachers differed considerably in professional experience (Classroom 1: 3.5 years; Classroom 2: 9.5 years). This difference is a potential confounding variable, as teaching experience may influence technique selection and efficacy. Although teacher identity was included as a random effect in all models to account for between-teacher variation, this does not eliminate the confound and is acknowledged as a limitation.

Data collection spanned 96 classroom hours. This was divided between two researchers, with one researcher typically assigned to each classroom. While researchers aimed to remain with their designated classes, the movement of both staff and students between rooms required flexibility, and incidents were recorded from either class as they arose. Although observations primarily took place in the two classrooms, additional incidents were recorded in communal areas such as the playground during break times.

#### Inclusion/exclusion criteria and consent

Participants were included if they were enrolled in one of the two target classrooms for the duration of the study and had provided consent. No children or teachers were excluded. Prior to the commencement of data collection, written informed consent was obtained from teachers as well as from parents or legal guardians of the children.

### Procedure

#### Researcher role and familiarisation

To minimise observer effects and disruption to classroom routines, researchers spent 1 week in the classrooms before formal data collection began. During this familiarisation period, they were introduced to the children as university students learning about effective teaching practices. Researchers participated in classroom activities and play, helping to normalise their presence and the use of note-taking.

Prior to formal data collection, both researchers independently coded a set of pilot incidents from the familiarisation week and then met to compare ratings and resolve discrepancies. This calibration process was repeated twice during the data collection period to identify and correct any emerging interpretive drift between coders.

This approach.was intended to reduce the likelihood that teachers’ or students’ behaviour would be altered by observation; however, observer effects and the Hawthorne effect (McCarney et al., 2007) cannot be fully eliminated and may have influenced some interactions. While the teaching staff were fully briefed on the aims of the study, specific details of the observational focus were withheld to minimise potential bias.

#### Data collection

During observations, researchers adopted a stance of positive neutrality: they remained friendly and approachable to students while deferring all authority to the teaching staff. Boundaries were strictly maintained, with researchers refraining from involvement in incident management to minimise any influence on behaviour.

Data were collected on average 3 days per week, scheduled to align with periods of maximum student attendance. To ensure consistency and minimise disruption, researchers were present from before student arrival until lunchtime. Data collection was therefore limited to morning sessions, which constitutes temporal sampling. Post-lunch periods, which may be associated with heightened dysregulation following transitions or afternoon fatigue, were not systematically captured. This is acknowledged as a limitation: the dataset may under-represent certain types or times of dysregulation incidents. A dual data collection method was employed: discreet audio recordings captured interactions verbatim, while typed notes recorded non-verbal cues, gestures, and contextual details. Students were unaware of the audio recording, and if questioned, researchers explained that they were *taking study notes*. This approach aimed to preserve a naturalistic classroom environment and reduce external influence on behaviour.

#### After observation procedure

Following each observation session, researchers reviewed and synthesised the data. Audio recordings were transcribed and cross-referenced with observational notes to produce comprehensive verbatim accounts of incidents. This integration of verbal and non-verbal information was critical for interpreting context. For example, the phrase *Are you tired?* spoken in a calm tone may represent a teacher’s attempt to interpret a child’s inner state, whereas the same phrase delivered accusatorily may induce fear and insecurity.

Each incident transcript was entered into a spreadsheet detailing key information, including the teacher and child involved. Data were cross-checked for integrity, with each researcher’s work reviewed before being merged into a master database.

Coding of the data proceeded in two stages. First, each teacher–student interaction was coded using a predefined mentalization framework. Second, interactions were assigned an *Interaction Severity* rating, while each incident was given an *Overall Incident Severity* score based on the highest-rated interaction within it. Additional coding was conducted beyond the scope of this study. Following coding, both quantitative and qualitative analyses were performed.

#### Ethical considerations

Confidentiality was maintained at all times. All names of students and teachers were anonymised in transcripts and data files. Audio recordings and electronic data were stored in a secure, password-protected database. To safeguard participant privacy, all raw data, including audio recordings, will be permanently deleted in accordance with data protection guidelines. The data collection procedure was approved by UCL’s Research Ethics Committee in accordance with the BPS Code of Human Research Ethics (BPS, 2021). Written consent was obtained from parents and guardians, and students were debriefed at the close of the study.

### Measures

The data collected for this study included the time, cohort, trigger, behaviour, initial teacher response, child response, and subsequent teacher responses until the incident was resolved or concluded. During coding, two measures of severity were recorded alongside the category of mentalization strategies and the specific mentalization techniques used. Additional variables were coded for a separate study but are not included in the present analysis.

#### Incident recording

**Figure 1:**
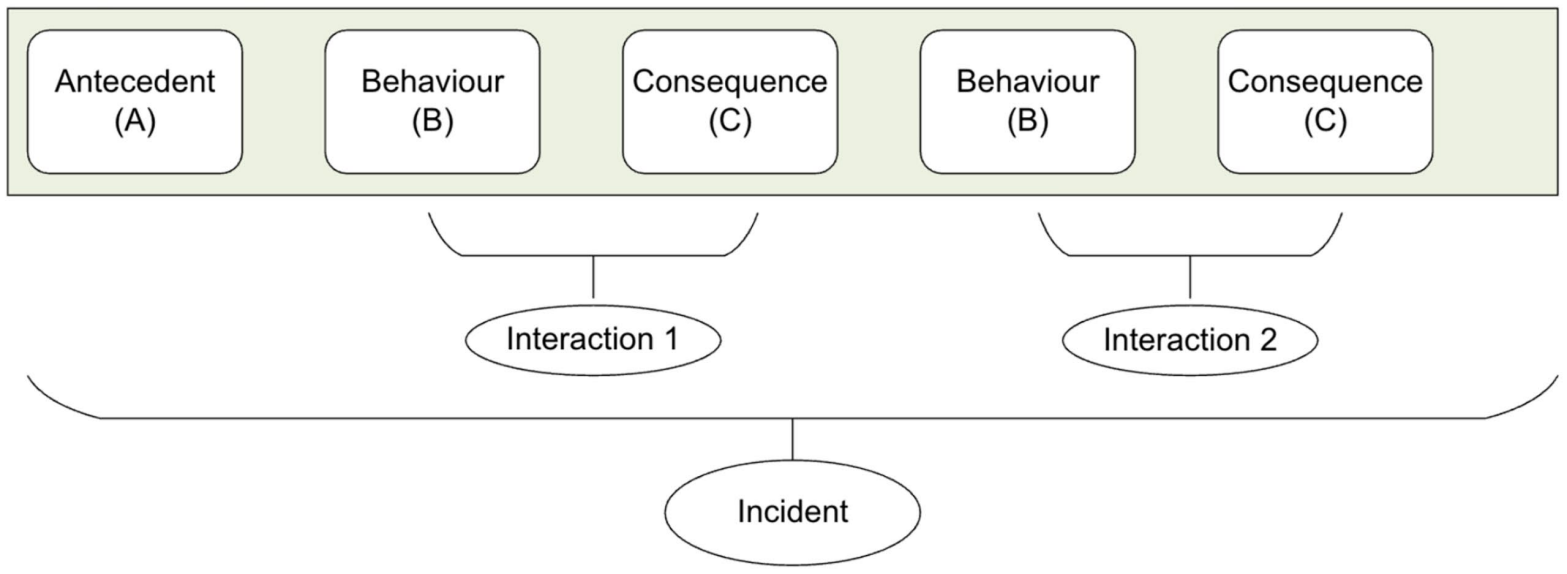
ABC incident model example of a two-interaction incident. This model breaks down an incident into its core components: antecedent (A), behaviour (B), and consequence (C). The diagram shows an incident composed of two interactions.

For the purposes of analysis, an *incident* was defined as the full sequence of dialogue – verbal and non-verbal – and the interaction between a teacher and a child in relation to a specific episode of disruptive behaviour or emotional dysregulation. Each incident consisted of one or more *interactions*, with an interaction defined as a single exchange between teacher and child (see Figure 1 for an example).

The observational record used was a researcher-developed instrument adapted from the Antecedent–Behaviour–Consequence (ABC) incident model, and consistent with the data collection approach described in Chelouche-Dwek et al. (2025). Incidents were structured for analysis using the model, first conceptualised by Bijou and Baer (1961) and rooted in the three-term contingency framework (Skinner, 1965). This model was chosen because it provides a clear sequence of events around challenging behaviour, helping to identify triggers (antecedents) as well as responses (Meadan et al., 2016). Behaviour was defined as any verbal or non-verbal response from the child, while the consequence was defined as the teacher’s immediate response, aimed at remediation. The ABC model was retained not as a theoretical commitment but as a practical organisational scaffold that provides a consistent, transparent structure for recording the sequence of events within each incident. The mentalization coding layer, applied after the ABC record was complete, is the analytic vehicle through which internal states and relational processes are examined.

An incident was considered closed when one of the following criteria was met: (a) the child returned to pre-incident baseline behaviour (e.g., resumed on-task activity, ceased the disruptive behaviour); (b) a natural environmental event ended the exchange (e.g., a school bell, a transition); or (c) more than 5 min elapsed without further teacher–child interaction related to the same triggering event.

Following a consequence, each interaction was coded as either *resolved* or *unresolved*. If resolved, the incident was closed. If unresolved, subsequent child behaviours and teacher responses were recorded, with this cycle repeating until the incident was either resolved or concluded by other means. For example, an unresolved conclusion might occur if a child stops shouting not because of the teacher’s intervention but because the bell rings for break. An example of a recorded incident is provided in Table A.

#### Data coding

After data collection, each interaction within an incident was systematically coded. The primary coding fields for this study were the category of mentalization strategies and the specific mentalization technique used by the teacher. Incident severity was also coded in two ways to provide contextual information. An example of a coded incident is provided in Table B.

#### Severity

To assess whether an incident improved or deteriorated across interactions, a measure of interaction severity was applied. This allowed for tracking progress even in cases where incidents were not fully resolved. Interaction severity was rated on a three-point ordinal scale:

1. *Level 1 behaviours* were low-level disruptions, such as being off-task, failing to follow instructions, or making distracting noises.
2. *Level 2 behaviours* included Level 1 behaviours accompanied by negative intent, such as name-calling, deliberate refusal to comply, or intentionally distracting peers.
3. *Level 3 behaviours* were the most serious, encompassing repeated Level 2 actions, dangerous behaviours (including aggression or throwing objects), discriminatory actions, and overt defiance.

In addition, each incident was assigned an *Overall Incident Severity* score, corresponding to the highest severity level reached at any point. This provided a metric for evaluating whether different techniques were effective in resolving incidents of varying peak severities.

#### Coding frame for mentalization categories and techniques

The type and frequency of mentalization techniques used by teachers were categorised using a structured coding frame adapted from the framework developed by Muñoz Specht et al. (2016) for psychodynamic therapy. Their framework incorporated established mentalization techniques from manuals and articles on interventions for children and adolescents. Two key sources were the book chapter *Mentalization-Based Treatment for Adolescents with Borderline Traits* (Fonagy et al., 2014) and the adult MBT manual *Mentalizing in Clinical Practice* (Allen et al., 2008). For the purposes of this study, the framework was streamlined by merging categories with overlapping themes to suit the educational context.

The adaptation of the Muñoz Specht et al. (2016) framework proceeded in three stages. First, all original techniques were reviewed for applicability to an educational (rather than therapeutic) context. Second, techniques whose operational definitions became overlapping when applied to classroom interactions were merged; for example, techniques concerned with monitoring and supporting the mentalizing stance were combined into a single stabilising category. Third, a pilot coding exercise was conducted: both researchers independently coded 20 incidents from the familiarisation week using the adapted framework, then compared and discussed ratings until consensus was reached on category boundaries. Only after this process was the 13-technique framework finalised.

Each interaction was coded on a scale from 0 to 3. A code of *0* was applied when no mentalizing technique was evident, typically involving direct commands. When mentalization techniques were identified, they were classified into one of three categories: *Supporting the Stabilising Stance*, *Basic Mentalizing Techniques*, or *Contextualising the Learning Environment*.

Inter-rater reliability for both the mentalization technique coding and the severity scale was formally assessed using Cohen’s Kappa; results are reported in the Quantitative Results section.

##### Supporting the stabilising stance

This category included techniques that create the conditions necessary to engage a child’s mentalizing capacity. These involved clarifying rules and boundaries, building trust, and establishing mutual understanding. It also included empathic responses designed to help regulate a child’s emotions, a strategy frequently used in MBT (Fonagy et al., 2014), validating the child’s feelings as a precursor to deeper mentalizing.

##### Basic mentalizing techniques

These were core interventions aimed at directly enhancing a child’s capacity for mentalization. In the classroom, they included adopting a *not-knowing* stance, emphasised by Allen et al. (2008), characterised by curiosity and openness. Teachers also used these techniques to highlight the perspectives of others (e.g., considering what classmates or other staff might think), thereby fostering empathy and situating the child within a broader social context. Additionally, they encompassed moments when teachers tentatively connected ideas to a child’s beliefs, encouraging reflection.

##### Contextualising the learning environment

This category included interventions that raised awareness of mental states through broader, more abstract strategies. Teachers drew attention to dissociative reactions, explored situational or relational contexts, and helped children make sense of their environment. It also included recognising and affirming a child’s progress in self-regulation, in line with government guidance promoting positive behaviour (Department for Education, 2024).

Across these three categories, the framework was operationalised into 13 specific mentalization techniques (see Table 1), each accompanied by a description and example of its use in an educational context.

**Table 1:** Coding frame for teacher mentalization categories and techniques.

Mentalization category	Technique	Brief description	Teacher example
Supporting the stabilising stance	1. Classroom framework interventions	Clarifying structure, expectations, and boundaries of the classroom to support regulation.	“Let us remember our calm-down plan. We have our breathing space and our fidget tools available.”
	2. Supportive and empathic interventions	Expressing empathy and validation to support emotional containment.	“I can see you are really upset right now. Those are big feelings, and it makes sense you are having them.”
	3. Clarification and exploration	Using open-ended questioning to clarify and explore the child’s experience.	“Can you help me understand what’s happening for you right now?”
Basic mentalizing techniques	4. Exploring mental states of self	Encouraging the child to reflect on their own feelings, thoughts, and bodily sensations.	“What’s your body telling you right now? Are your shoulders tight? Is your heart beating fast?”
	5. Exploring mental states of others	Helping the child consider others’ perspectives and feelings.	“I wonder how she felt when you walked away from the group. What do you think was going through her mind?”
	6. Exploring mental states in relationships	Promoting reflection on interactions with peers or the teacher during dysregulation.	“It seems like when I asked you to transition, something shifted between us. What was that like for you?”
	7. Addressing contradictions	Gently pointing out contradictions to support the integration of conflicting feelings.	“You said you do not care about the project, but I noticed you spent a lot of time on your drawing yesterday. I’m wondering if maybe part of you does care?”
	8. Interpretive connecting	Making tentative connections that link behaviour with underlying feelings or needs.	“I’m wondering if when you pushed the materials away, maybe you were feeling overwhelmed and needed some space?”
	9. Teacher’s own mental states	Sharing aspects of the teacher’s own thoughts or feelings to model reflective thinking.	“I’m feeling a bit confused right now because I want to help, but I’m not sure what you need from me.”
Contextualising the Learning Environment	10. Clarifying the situation narrative	Helping the child articulate what happened in a clear and coherent way.	“Let us walk through what happened step by step. First, you were working on maths, then what came next?”
	11. Exploring classroom relationships	Examining dynamics between the child and classmates during dysregulation.	“It seems like something happened between you and your table group that was upsetting. Can we talk about what that was like?”
	12. Understanding situational context	Focusing on the meaning of classroom situations that triggered dysregulation.	“The fire drill was really loud and unexpected. For some people, sudden loud noises can feel scary or overwhelming. Was that your experience?”
	13. Highlighting progress in regulation	Drawing attention to improvements or positive changes in how the child manages emotions.	“I noticed that even though you were really upset, you used your words to tell me instead of throwing something. That’s growth!”

Coding frame adapted from Muñoz Specht et al. (2016).

### Data analysis

#### Quantitative analysis

The quantitative analysis was carried out in three stages. First, the reliability of the coded data was established. Second, the effectiveness of mentalization categories was compared with non-mentalizing approaches. Finally, the performance of individual mentalizing techniques was examined.

##### Inter-rater reliability

To evaluate coding consistency, inter-rater reliability was assessed using Cohen’s Kappa, which ranges from −1 to +1, with values closer to +1 reflecting stronger agreement. Following the guidelines of Landis and Koch (1977), a threshold of 0.8 was set to indicate strong agreement. Kappa was calculated for both the mentalization categories and the specific techniques, based on coding conducted by two independent raters. This step ensured the coding was consistent and reliable before progressing to further analyses.

##### Analysis overview

An initial descriptive overview of the dataset was conducted to provide context. This included examining the total number of interactions and incidents, along with the mean and median incident lengths, to establish the basic structure of the data.

The primary analysis used cross-categorisation models to account for the complex, non-hierarchical nature of the data, where incidents were not neatly nested within students or teachers. This modelling choice better reflected the reality that both students and teachers contribute to incident resolution (Goldstein, 2011; Raudenbush, 1993). A cross-categorisation model was selected specifically because the data structure violated the assumptions of simple hierarchical multilevel models: the same student could interact with multiple staff members, and the same teacher responded to multiple students across incidents. Cross-categorisation models address this by treating student, teacher, and incident as crossed (rather than nested) random effects, simultaneously accounting for all three sources of clustering without assuming that one is contained within another (Goldstein, 2011; Raudenbush, 1993). This approach more accurately reflects the relational reality of the classroom. Two models were run to assess the impact of mentalization categories, and two further models were used to examine the effects of individual mentalizing techniques.

In all four analyses, three random effects were included: teacher, child, and incident. Teacher accounted for individual teaching style and experience, child controlled for variability in how often individual children were involved in incidents, and incident addressed the multiple resolution attempts within a single episode. Although classroom membership was recorded, it was not included as a random effect because both teachers and students often moved between classrooms.

Incident severity and interaction severity were also included in the models to test whether severity predicted resolution and to control for its potential influence on the effectiveness of interventions.

Results were reported as odds ratios (OR) with corresponding 95% confidence intervals and *p*-values, with significance set at *p* < 0.05. An OR above 1 indicated a higher likelihood of resolution compared to the reference category, while an OR below 1 indicated a lower likelihood. Model fit was evaluated using the F-statistic and its associated p-value. If severity emerged as a significant predictor, a supplementary analysis of the interaction between mentalization techniques and severity was planned to assess whether particular techniques were more effective in resolving high-severity incidents.

##### Effectiveness of mentalization categories

The first analysis compared the three mentalization categories with the non-mentalizing category to test whether any mentalizing approach was significantly more effective in resolving incidents. A second analysis set Category 1, *Supporting the Stabilising Stance*, as the baseline. Categories 2 (*Basic Mentalizing Techniques*) and 3 (*Contextualising the Learning Environment*) were compared against this baseline to assess whether the use of more complex strategies conferred greater efficacy.

##### Effectiveness of mentalization techniques

A similar two-step analysis was conducted for the 13 individual techniques. First, all techniques were compared against a non-mentalizing baseline. Second, the analysis was repeated using Technique 1, *Classroom framework interventions*, as the baseline. This technique, which focuses on rules and structure (Muñoz Specht et al., 2016), was chosen because it represents a more foundational and less explicitly interactive form of mentalizing. Using this as the comparator allowed for an evaluation of whether more complex or explicitly mentalizing techniques outperformed this basic, structural approach.

#### Qualitative analysis

Following the completion of the quantitative analyses, a sequential and integrated process was adopted. The statistical findings informed the subsequent thematic analysis of the qualitative data, allowing for a focused exploration of the patterns observed in the quantitative results (Creswell and Plano Clark, 2017). The purpose was to clarify and extend the earlier findings, supporting the discussion with context-rich examples.

Particular attention was given to techniques that performed substantially worse than the baseline or markedly better than other techniques. Alongside this, a general overview of the three mentalizing categories was developed, with consideration also given to some aspects of non-mentalizing techniques. This dual focus provided both a broad overview of technique uses, and a closer examination of how therapeutic techniques translated into the educational setting.

A thematic analysis was conducted following the approach of Braun and Clarke (2006). All incident transcripts were analysed, including verbal and non-verbal dialogue. The pre-identified mentalization techniques served as overarching codes to structure the analysis. Key ideas and recurring patterns associated with each strategy and category were then categorised and annotated using Atlas.ti 23 software. This approach is a theory-driven, deductive thematic analysis, differing from a reflexsive thematic analysis as described in Braun and Clarke (2006) later work. The analysis uses pre-specified codes (the 13 techniques) as *a priori* organising categories with an explanatory analytic goal. This was to determine why certain techniques performed as they did, rather than theory-generative. This process enabled the identification of context-specific dynamics in the data. The transcript excerpts presented in the qualitative findings section were selected for their illustrative quality: they exemplify the key features of a given technique as identified across the full dataset, rather than as statistically representative samples. They were selected collaboratively after coding was complete.

##### Integrity

The qualitative analysis was undertaken after establishing the reliability of the coding frame, which increased confidence in using this pre-determined framework to interpret the data. This contributed to consistency across the coding of all 815 interactions. Regular meetings were held between coders throughout the process to resolve discrepancies, ensuring a shared interpretative stance and reinforcing the dependability of the analysis.

##### Reflexivity

Both researchers were graduate students trained in mentalization theory, which inevitably shaped what they noticed, recorded, and interpreted. Their direct presence in the classrooms may have enriched contextual understanding but also introduced the risk of confirmatory interpretation. To mitigate this, all coding was conducted after observations had ended; coding categories were operationally defined before analysis began; inter-rater reliability was formally assessed; and discrepancies were resolved through structured discussion. The qualitative interpretations should be understood as theory-informed rather than theory-neutral.

The qualitative analysis also created an opportunity to consider reinforcement strategies used by teachers once an incident was resolved. These included techniques aimed at encouraging positive behaviour or recognising progress in emotional regulation. Although excluded from the main analysis because they occur after incident resolution, these reinforcement strategies are discussed separately as supplementary material to provide a fuller picture of the classroom dialogue.

## Results

### Quantitative results

#### Inter-rater reliability

To confirm coding consistency, inter-rater reliability was assessed using Cohen’s Kappa. The analysis indicated strong agreement between raters for the coding of mentalization categories, with a kappa coefficient of 0.93 (*p* < 0.001). Agreement for the coding of individual techniques was also strong, with a kappa coefficient of 0.94 (*p* < 0.001).

Both coefficients exceeded the 0.8 threshold for strong agreement, confirming a high level of consensus between raters. These results support the reliability of the coding system and the validity of the subsequent analyses.

#### Overview of data

**Figure 2:**
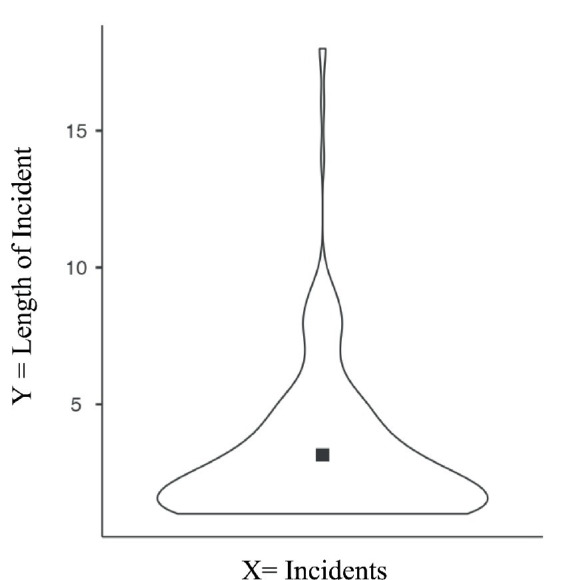
A violin plot illustrating the lengths of incidents. The violin plot illustrates the distribution of incident lengths. Mean is also denoted (M = 3.15).

Descriptive analyses provided an overview of the dataset (Table 2). A total of 815 teacher–child interactions were recorded, grouped into 259 incidents. On average, incidents involved just over three interactions before resolution (*M* = 3.15, *SD* = 2.68), with a median length of two interactions. This indicates a positively skewed distribution, indicating that the majority of incidents involved a small number of interactions. However, a brief incident does not necessarily indicate successful resolution – some short incidents may have concluded without full resolution due to an external interruption (e.g., a school bell). The distribution reflects the varying duration of dysregulation episodes rather than uniformly successful rapid resolution. There were a smaller number of longer incidents requiring extended exchanges, the largest containing 18 interactions. This distribution, along with the mean, is illustrated in the violin plot in Figure 2.

**Table 2:** Descriptive statistics.

Measure	Incident number
*N* (Incident)	259
*N* (Interaction)	815
Mean	3.15
Median	2
Standard deviation	2.68
Minimum	1
Maximum	18

The descriptive statistics refer to the lengths of incidents.

Variance component estimates from the cross-categorisation model indicated moderate between-teacher variability (σ^2^ = 0.174, SE = 0.136, 95% CI [0.038, 0.802], *p* = 0.200) and comparable between-student variability (σ^2^ = 0.177, SE = 0.134, 95% CI [0.040, 0.782], *p* = 0.188). Incident-level variance was negligible (σ^2^ = 1.17 × 10^−6^, SE = 4.69 × 10^−6^, 95% CI [4.60 × 10^−10^, 0.003], *p* = 0.803), suggesting that residual variability was primarily attributable to differences between teachers and students rather than incident-specific factors. None of the variance components reached statistical significance, which is consistent with the small sample sizes and wide confidence intervals noted elsewhere in the Results.

#### Effectiveness of mentalizing on incident resolution

A cross-categorisation model was employed to examine how different mentalizing categories influenced the likelihood of incident resolution. The model controlled for incident severity and included random effects for students, teachers, and incidents.

The overall model was statistically significant, *F*(5, 809) = 24.05, *p* < 0.001, indicating that the predictor variables reliably accounted for incident resolution (Table 3). Significant main effects were found for incident severity, *F*(2, 809) = 9.92, *p* < 0.001, and for the mentalizing category used, *F*(3, 809) = 33.03, *p* < 0.001 (Table 3). Akaike’s Information Criterion (AIC) = 4103.4. To assess specific effects, odds ratios (*OR*) were calculated (Table 4).

**Table 3:** Overall model including incident severity and effectiveness of mentalization categories.

Source	*F*	*df1*	*df2*	*p*
Corrected Model	24.048	5	809	<0.001
OverallSeverity123	9.922	2	809	<0.001
Which123	33.025	3	809	<0.001

Target: Resolved? Yes/No.

**Table 4:** Cross-categorisation model comparing incident severity and effectiveness of mentalization categories.

Model term	Coefficient	Std. error	*t*	*p*	95% CI	Exp (coefficient)	95% CI for Exp (coefficient)
Intercept	−1.360	0.3516	−3.868	<0.001	[−2.050, −0.670]	0.257	[0.129, 0.512]
Overall Severity 123 = 3	−1.234	0.2885	−4.278	<0.001	[−1.800, −0.668]	0.291	[0.165, 0.513]
Overall Severity 123 = 2	−0.531	0.2813	−1.889	0.059	[−1.083, 0.021]	0.588	[0.338, 1.021]
Overall Severity 123 = 1	$0^{b}$	-	-	-	-	-	-
which123 = 3	2.551	0.3108	8.209	<0.001	[1.941, 3.161]	12.825	[6.968, 23.605]
which123 = 2	2.104	0.2534	8.306	<0.001	[1.607, 2.602]	8.202	[4.988, 13.486]
which123 = 1	1.483	0.2536	5.846	<0.001	[0.985, 1.980]	4.404	[2.677, 7.245]
which123 = 0	$0^{b}$	-	-	-	-	-	-

“b” means the baseline category.

##### Incident severity

Level 1 severity was used as the reference category. Compared with this baseline, Level 3 severity was associated with a significant reduction in resolution likelihood (*OR* = 0.29, 95% CI [0.17, 0.51], *p* < 0.001). The effect of Level 2 severity was not statistically significant (*p* = 0.059).

##### Mentalizing strategies

Each of the three mentalizing categories was significantly more effective than non-mentalizing strategies in resolving incidents. Supporting the Mentalizing Stance (Category 1) was over four times more likely to lead to resolution compared with non-mentalizing approaches (*OR* = 4.40, 95% CI [2.68, 7.25], *p* < 0.001). Basic Mentalizing Techniques (Category 2) increased the odds of resolution by more than eight times (OR = 8.20, 95% CI [4.99, 13.49], *p* < 0.001). Contextualising the Learning Environment (Category 3) proved most effective, increasing the odds of resolution nearly thirteen-fold (*OR* = 12.83, 95% CI [6.97, 23.61], *p* < 0.001).

#### Comparing effectiveness of mentalizing categories

Since all three mentalizing categories significantly outperformed non-mentalizing approaches, a further cross-categorisation model compared the categories against one another. Category 1 (Supporting the Mentalizing Stance) was set as the baseline because it represents the least complex set of interventions. The model also controlled for interaction severity and included random effects for teacher, child, and incident. The overall model was statistically significant, *F*(4, 350) = 4.62, *p* = 0.001 (Table 5).

**Table 5:** Overall model including interaction severity and comparison between mentalization categories.

Source	*F*	*df1*	*df2*	*p*
Corrected Model	4.622	4	350	0.001
Severity123	3.691	2	350	0.026
Which123	6.544	2	350	0.002

Target: Resolved? Yes/No.

##### Interaction severity

The findings mirrored those of overall incident severity. A significant main effect was observed, *F*(2, 350) = 3.69, *p* = 0.026. Using Level 1 as the baseline, Level 3 interactions were significantly less likely to be resolved (*OR* = 0.45, 95% CI [0.25, 0.83], *p* = 0.011). No significant effect was observed for Level 2 severity (*p* = 0.64). These results suggest that only the most severe interactions significantly reduce the likelihood of resolution (Table 6).

**Table 6:** Cross-categorisation model comparing interaction severity and comparison between mentalization categories.

Model term	Coefficient	Std. error	*t*	*p*	95% CI	Exp (coefficient)	95% CI for Exp (coefficient)
Intercept	−0.527	0.3625	−1.454	0.147	[−1.240, 0.186]	0.590	[0.289, 1.204]
Severity 123 = 3	−0.795	0.3097	−2.565	0.011	[−1.404, −0.185]	0.452	[0.246, 0.831]
Severity 123 = 2	−0.132	0.2812	−0.47	0.639	[−0.685, 0.421]	0.876	[0.504, 1.523]
Severity 123 = 1	$0^{b}$	-	-	-	-	-	-
which123 = 3	1.088	0.3124	3.482	<0.001	[0.473, 1.702]	2.967	[1.605, 5.485]
which123 = 2	0.608	0.2593	2.346	0.02	[0.098, 1.118]	1.837	[1.103, 3.060]
which123 = 1	$0^{b}$	-	-	-	-	-	-

Target: Resolved? Yes/No. Baseline categories are set to 0. “b” means the baseline category.

##### Mentalizing categories

The effect of mentalizing category was highly significant, *F*(2, 350) = 6.54, *p* = 0.002. Compared to Category 1, Basic Mentalizing Techniques (Category 2) increased the odds of resolution by a factor of 1.84 (*OR* = 1.84, 95% CI [1.10, 3.06], *p* = 0.02). Contextualising the Learning Environment (Category 3) was even more effective, nearly tripling the odds of resolution (*OR* = 2.97, 95% CI [1.61, 5.49], *p* < 0.001). These findings suggest a graded effect in which more complex mentalization strategies are progressively more effective.

#### Analysis of interaction effects

Because both measures of severity – individual interaction and overall incident – were significant predictors alongside mentalizing categories, a supplementary exploratory analysis was conducted. The aim was to test for interaction effects to determine whether the effectiveness of a mentalizing technique depended on the severity of the incident.

The analysis revealed no statistically significant interaction between incident or interaction severity and mentalizing techniques. This null result should be interpreted with caution: it does not constitute evidence that such interactions do not exist in the population. Given the small number of high-severity incidents involving any specific technique, the study was underpowered to detect technique-by-severity interactions. This question warrants investigation in future studies with larger samples.

#### Effectiveness of specific mentalizing techniques

A cross-categorisation model was used to compare the effectiveness of the 13 specific techniques against a baseline of non-mentalizing approaches. The model controlled for interaction severity and included random effects for child, teacher, and incident. The overall model was statistically significant, *F*(15, 799) = 9.20, *p* < 0.001, with a strong main effect for specific technique, *F*(13, 799) = 10.29, *p* < 0.001. Interaction severity was not significant (*p* = 0.051) (Table 7).

**Table 7:** Overall model including interaction severity and effectiveness of mentalization techniques.

Source	*F*	*df1*	*df2*	*p*
Corrected Model	9.199	15	799	<0.001
Severity123	2.990	2	799	0.051
specific	10.290	13	799	<0.001

Target: Resolved? Yes/No.

The confidence intervals for several technique-level effects are wide, reflecting the small number of incidents coded with each specific technique. Wide CIs indicate substantial uncertainty in the point estimates; the true population effect size may differ considerably from the observed value, and results should be interpreted accordingly.

When each technique was compared with the non-mentalizing baseline, most were significantly more effective at achieving resolution, although two were not (Table 8).

**Table 8:** Cross-categorisation model comparing interaction severity and effectiveness of mentalization techniques.

Model term	Coefficient	Std. error	*t*	*p*	95% CI	Exp (coefficient)	95% CI for Exp (coefficient)
Intercept	−1.941	0.3072	−6.317	<0.001	[−2.544, −1.338]	0.144	[0.079, 0.262]
Severity123 = 3	−0.55	0.272	−2.02	0.044	[−1.083, −0.016]	0.577	[0.338, 0.984]
Severity123 = 2	0.043	0.2467	0.174	0.862	[−0.441, 0.527]	1.044	[0.643, 1.694]
Severity123 = 1	$0^{b}$	-	-	-	-	-	-
specific = 13	2.634	0.5174	5.09	<0.001	[1.618, 3.650]	13.927	[5.044, 38.457]
specific = 12	2.683	0.696	3.854	<0.001	[1.316, 4.049]	14.623	[3.730, 57.328]
specific = 11	2.678	0.4391	6.099	<0.001	[1.816, 3.540]	14.553	[6.147, 34.456]
specific = 10	2.464	0.5804	4.245	<0.001	[1.324, 3.603]	11.749	[3.760, 36.712]
specific = 9	1.256	0.447	2.81	0.005	[0.379, 2.133]	3.511	[1.460, 8.444]
specific = 8	2.764	0.6159	4.488	<0.001	[1.555, 3.973]	15.871	[4.737, 53.169]
specific = 7	−0.162	1.0818	−0.15	0.881	[−2.286, 1.961]	0.85	[0.102, 7.109]
specific = 6	3.183	0.5429	5.862	<0.001	[2.117, 4.248]	24.111	[8.305, 69.998]
specific = 5	3.16	0.4853	6.511	<0.001	[2.208, 4.113]	23.577	[9.093, 61.128]
specific = 4	2.22	0.4298	5.166	<0.001	[1.376, 3.064]	9.208	[3.961, 21.406]
specific = 3	0.743	0.4968	1.495	0.135	[−0.233, 1.718]	2.101	[0.792, 5.572]
specific = 2	2.388	0.4134	5.776	<0.001	[1.576, 3.199]	10.888	[4.836, 24.511]
specific = 1	1.459	0.3045	4.792	<0.001	[0.861, 2.057]	4.303	[2.367, 7.823]
specific = 0	$0^{b}$	-	-	-	-	-	-

Target: Resolved? Yes/No. Baseline categories are set to 0.

##### Non-significant mentalizing techniques

Two techniques were not statistically different from non-mentalizing approaches: *Clarification and Exploration* (Technique 3), *OR* = 2.10, 95% CI [0.79, 5.57], *p* = 0.135; and *Addressing Contradictions* (Technique 7), *OR* = 0.85, 95% CI [0.10, 7.11], *p* = 0.881.

##### Significant techniques

In contrast, eleven techniques significantly increased the odds of resolution. Three demonstrated particularly strong effects: *Exploring Mental States in Relationships* (Technique 6), *OR* = 24.11, 95% CI [8.31, 70.00], *p* < 0.001; *Exploring Mental States of Others* (Technique 5), *OR* = 23.58, 95% CI [9.09, 61.13], *p* < 0.001; and *Interpretive Connecting* (Technique 8), *OR* = 15.87, 95% CI [4.74, 53.17], *p* < 0.001. Techniques 5 and 6, notably, were around 24 times more effective than non-mentalizing techniques in resolving incidents.

The remaining eight techniques also demonstrated statistically significant positive effects (all *p*s ≤ 0.005). These included: *Classroom Framework Interventions* (Technique 1), *Supportive and Empathic Interventions* (Technique 2), *Exploring Mental States of Self* (Technique 4), *Teachers’ Own Mental States* (Technique 9), *Clarifying the Situation Narrative* (Technique 10), *Exploring Classroom Relationships* (Technique 11), *Understanding Situational Context* (Technique 12), and *Highlighting Progress in Regulation* (Technique 13).

#### Comparative efficacy of mentalizing techniques

To compare the relative effectiveness of techniques, a second model used *Classroom Framework Interventions* (Technique 1) as the baseline. This technique was chosen because it represents a foundational form of mentalizing, providing structure and rules while requiring less use of theory of mind than more advanced strategies. The model controlled for interaction severity (a significant predictor, *p* = 0.027) and included random effects for child, teacher, and incident. The overall model was statistically significant, *F*(14, 340) = 3.27, *p* < 0.001, with a strong main effect for technique, *F*(12, 340) = 3.44, *p* < 0.001 (Table 9).

**Table 9:** Overall model including interaction severity and comparison between mentalization techniques.

Source	*F*	*df1*	*df2*	*p*
Corrected Model	3.270	14	340	<0.001
Severity123	3.635	2	340	0.027
specific	3.436	12	340	<0.001

Target: Resolved? Yes/No.

##### Analysis of specific techniques

Several techniques; 13, 11, 2, 5, and 6; were significantly more effective than the baseline. Of these, two were particularly effective. *Exploring Mental States of Others* (Technique 5) increased the odds of resolution more than fivefold (*OR* = 5.52, 95% CI [1.96, 15.54], *p* = 0.001). Similarly, *Exploring Mental States in Relationships* (Technique 6) was strongly significant, increasing the odds of resolution by a factor of five (*OR* = 5.02, 95% CI [1.57, 16.02], *p* = 0.007) (Table 10).

**Table 10:** Cross-categorisation model comparing interaction severity and comparison between mentalization techniques.

Model term	Coefficient	Std. error	*t*	*p*	95% CI	Exp (coefficient)	95% CI for Exp (coefficient)
Intercept	−0.713	0.4493	−1.587	0.114	[−1.597, 0.171]	0.49	[0.203, 1.186]
Severity123 = 3	−0.798	0.3414	−2.339	0.02	[−1.470, −0.127]	0.45	[0.230, 0.881]
Severity123 = 2	−0.005	0.3154	−0.017	0.986	[−0.626, 0.615]	0.995	[0.535, 1.850]
Severity123 = 1	$0^{b}$	-	-	-	-	-	-
specific = 13	1.226	0.5533	2.215	0.027	[0.137, 2.314]	3.406	[1.147, 10.115]
specific = 12	1.299	0.7516	1.729	0.085	[−0.179, 2.778]	3.667	[0.836, 16.081]
specific = 11	1.179	0.4832	2.44	0.015	[0.228, 2.129]	3.251	[1.257, 8.408]
specific = 10	1.048	0.6145	1.705	0.089	[−0.161, 2.256]	2.851	[0.851, 9.546]
specific = 9	−0.264	0.4892	−0.54	0.59	[−1.226, 0.698]	0.768	[0.293, 2.010]
specific = 8	1.227	0.6495	1.889	0.06	[−0.050, 2.504]	3.411	[0.951, 12.236]
specific = 7	−1.883	1.1082	−1.699	0.09	[−4.063, 0.297]	0.152	[0.017, 1.346]
specific = 6	1.613	0.5901	2.733	0.007	[0.452, 2.774]	5.018	[1.572, 16.019]
specific = 5	1.708	0.5265	3.244	0.001	[0.672, 2.744]	5.518	[1.959, 15.542]
specific = 4	0.848	0.4836	1.753	0.081	[−0.104, 1.799]	2.334	[0.902, 6.042]
specific = 3	−0.991	0.5474	−1.811	0.071	[−2.068, 0.086]	0.371	[0.126, 1.089]
specific = 2	0.959	0.4589	2.089	0.037	[0.056, 1.862]	2.609	[1.058, 6.433]
specific = 1	$0^{b}$	–	–	–	–	–	–

Target: Resolved? Yes/No. Baseline categories are set to 0. ^“*b*”^ means the baseline category.

These findings suggest that while many advanced techniques provide added benefit, those explicitly focused on exploring the mental states of peers and teachers (Techniques 5 and 6) demonstrate the greatest improvement over foundational strategies such as *Classroom Framework Interventions*.

### Qualitative analysis

This section presents the key findings from the thematic analysis of teacher–child interactions during incidents. It first provides an overview of the categories of mentalization techniques with illustrative examples, before turning to contextual insights that enrich the quantitative findings.

#### Supporting the stabilising stance

In this category, teachers employed techniques designed to support a child’s capacity to self-regulate. These interventions aimed to create a calm and safe environment in which the child could begin to de-escalate. For instance, teachers often used classroom frameworks to manage access to resources, such as: *When you are a bit more settled you can have it back* (Incident 243). While such interventions did not always resolve the incident immediately, they frequently reduced its severity and established a pathway toward resolution, often opening the door for further mentalization techniques.

##### Empathetic validation

Teachers also used supportive and empathetic interventions to validate a child’s emotional state without judgement. The purpose was to help the child feel seen and understood, which often served as a powerful de-escalation tool. For example: *I can tell you are frustrated. I completely understand why you are getting frustrated, but there’s nothing I can do to stop that right now* (Incident 133). By explicitly naming the child’s emotion and demonstrating understanding, the teacher validated the experience, showing that the child’s feelings were recognised and taken seriously, even when the underlying problem could not be resolved immediately.

##### Clarifying and exploratory questions

Teachers frequently asked clarifying and exploratory questions to gain insight into the child’s perspective. This technique modelled a *not-knowing stance*, signalling genuine curiosity about the child’s thought process. For instance: *What are you talking about, [Child]? Can you explain your logic on that, please? What have aliens got to do with it?* [calm tone] (Incident 236). In this example, the teacher’s curiosity interrupted the disruptive behaviour, encouraged the child to articulate their reasoning, and revealed a misunderstanding that diffused the situation.

#### Basic mentalizing techniques

Beyond stabilising strategies, teachers engaged in more active and interpretive mentalizing approaches. These involved explicitly discussing the mental states of the child, peers, or teachers to help construct coherent narratives of social situations. For example: *Is it because it looks like quite a big book and it’s a bit overwhelming? That’s fair enough* (Incident 96). Two further strategies were prominent: linking feelings to group goals and promoting self-reflection.

##### Linking feelings to actions and group goals

Here, teachers helped children connect their feelings and behaviours with the needs and goals of others, situating their actions within the shared classroom environment. For example: *So, [other child] wants to listen, and he wants to get to break on time. Do you want to get to break?* (Incident 105). By highlighting both the peer’s intention and a shared group goal, the teacher enabled the child to understand how their behaviour was preventing both themselves and their friend from reaching a desired outcome, thereby encouraging cooperation.

##### Promoting self-reflection

Teachers also promoted self-reflection by encouraging children to see themselves from an external perspective. This often involved gently contrasting the child’s positive self-concept with their negative behaviour, without shaming. For example: *Is that necessary? No, it’s not nice. It’s not kind. You’re a nice person, alright? But you are not saying nice things* (Incident 94). In this instance, the child’s dysregulation continued, yet the intervention highlighted the discrepancy between self and action, fostering awareness of how behaviour influences relationships.

#### Contextualising the learning environment

The most complex category of mentalizing techniques involved contextualising the learning environment. These abstract strategies moved beyond immediate behavioural management to draw the child’s attention to the broader context of their actions. For example: *Well done for asking politely and being respectful* (Incident 150). Such interventions often included clarifying situational narratives or recognising progress in self-regulation. By situating behaviour within the “bigger picture,” these techniques supported the development of social understanding and emotional intelligence.

##### Constructing event narratives

Teachers frequently supported children in co-constructing a narrative of an incident. Using open-ended questions and collaborative dialogue, they worked with the child to create a shared and more complete account of what had happened, incorporating multiple perspectives and clarifying misunderstandings. This process enabled children to make sense of complex social events. For example:

*(Incident 228)*

Teacher 1: *Are we checked out here [Teacher 2]?* [animated, playful tone].

Teacher 2: *I think we are* [animated, playful tone].

Teacher 1: *Alright. [Child’s name] well done! You did about 35 min of lesson!*

Teacher 2: *Yes, well done!* [animated, encouraging tone].

In this instance, two teachers co-constructed a positive narrative around a child’s classroom experience, using encouraging language and reframing the event in terms of achievement.

##### Highlighting progress and reinforcing regulation

Another contextualising strategy involved explicitly recognising a child’s self-awareness and regulation. These interactions often occurred after incidents had been resolved and were important for building a child’s sense of progress, reinforcing positive behaviour, and encouraging social growth.

A straightforward example was the use of simple, specific praise:

*(Incident 222) Great self-control there.*

Here, the teacher reinforced the specific skill of self-control, allowing the child to see themself as capable of managing their emotions.

In more complex exchanges, teachers reinforced children’s perspective-taking by highlighting acts of social consideration. For instance:

*(Incident 89)*

Child: *So, do you want the dark?*

Teacher: *That’s a good question! I’m okay with the dark, thank you for asking! Should we see what [other children] want?* [animated tone].

The teacher’s enthusiastic response: *That’s a good question! Thank you for asking!* affirmed the child’s effort to consider another’s perspective. By extending the question to include peers, the teacher encouraged the child to broaden their awareness of others’ mental states, deepening their social understanding.

#### Expanding key quantitative findings

The thematic analysis provides insight into why some techniques emerged as highly effective in the quantitative analysis, while others were less successful.

#### Exploring the mental states of others (technique 5)

This technique aimed to shift a child’s focus outward, de-centring their immediate emotions and engaging them in a pro-social role. Teachers invited children to consider others’ perspectives, prompting empathy and disrupting dysregulation.

##### Shifting perspective to a peer’s experience

*(Incident 131) You would not like it if someone took your things, so I cannot let you take [Child]‘s things when he’s not here.*

Here, the teacher encouraged perspective-taking by linking the behaviour to the child’s own likely feelings. This created an opportunity for the child to mentalize about the peer’s experience.

##### Providing pro-social alternatives

*(Incident 240) You have every right to ask [Child B] to stop singing, but instead of being like, ‘Stoooop, [Child B]!’, you can ask a lot more nicely, like, ‘Hey [Child B], could you please stop singing?’*

This response validated the child’s feelings – *You have every right* – while also modelling a socially appropriate alternative. It simultaneously preserved the child’s agency and taught a positive social skill.

#### Understanding mental states in relationships (technique 6)

This technique supported children in interpreting social interactions by linking actions with presumed thoughts and feelings. It offered guidance on potentially confusing exchanges and modelled constructive relational dynamics.

##### Explaining relational dynamics

*(Incident 161) You just focus on you and go back to your work. Reacting is going to make [Child B] want to come in more, so if you just ignore it, it might get a bit better.*

Here, the teacher explained the implicit relational logic of Child B’s behaviour, reframing it as a reaction-seeking strategy and offering a non-confrontational way to de-escalate.

##### Reframing intent and creating solutions

*(Incident 186) Okay, I think we can both do something to fix this. First of all, [Child A], how about you pick your stuff off the floor so that [Child B] does not confuse it for rubbish and he does not kick it.*

Instead of attributing negative intent to Child B, the teacher reframed the event as a misunderstanding – *confuse it for rubbish* – and moved the children toward collaborative problem-solving.

#### Addressing contradictions (technique 7)

This strategy was among the least effective, as it relied on logic during moments of heightened emotion. For many children, it came across as invalidating or accusatory, demanding a level of calm reflection unavailable in states of dysregulation.

##### Highlighting disconnects between words and actions

*(Incident 163) Can you come to your desk? You said it wasn’t a distraction, but it feels like that’s half the reason why you are walking around the classroom. Okay? Is that fair?*

Although logical, this approach risked being received as accusatory, escalating rather than resolving the behaviour.

##### Challenging global or defiant statements

*(Incident 165) Is that necessary? No, it’s not nice, it’s not kind. You’re a nice person, alright? But you are not saying kind things.*

The child’s dysregulated state may have rendered the logical challenge difficult to receive constructively – MBT theory suggests that when mentalizing has broken down, contradictions risk being experienced as confrontational rather than collaborative (Bateman et al., 2023). Whether any specific child experienced a given intervention as dismissive rather than enlightening cannot be determined from observational data alone, and this interpretation is offered as a theoretical hypothesis requiring verification through methods that access children’s subjective experience directly.

#### Clarification and exploration (technique 3)

This technique was also largely ineffective in resolving incidents, often because it was employed at the early stages of dysregulation, when children were least able to engage in reflective self-report.

##### Emergence of an incident

*(Incident 188)*

Teacher: *[approaches child] Are you feeling okay? What’s wrong* [soft tone].

Child: *No, I wanted cereal.*

Teacher: *You wanted cereal? Alright. Let us do a bit of English and then we can see. Alright?* [soft, calm tone].

Child: *Alright.*

Here, the exploration revealed the root cause of the incident, even though it did not directly resolve it. The subsequent supportive intervention carried the de-escalatory weight.

##### High cognitive demand

*(Incident 1)*

Child: *[Pushes a toy car into a wall.]*

Teacher: *What have you done there?*

Child: *[Pushes the car harder into the wall and hits it].*

The open-ended question demanded a level of reflection the child could not access in that moment. The question was perceived less as curiosity and more as reprimand, escalating the behaviour rather than resolving it.

#### Teacher’s own mental states (technique 9)

This technique involved teachers sharing aspects of their own mental states; their uncertainty, curiosity, or confusion, as a way of modelling reflective thinking and reducing the evaluative pressure that can inhibit a child’s own openness.

*(Incident 217)*

Teacher: *I’m feeling a bit confused right now, because I want to help you, but I’m not sure what you need from me.*

By making the teacher’s uncertainty explicit, this intervention repositioned the teacher as a co-thinker rather than an authority, which appeared to reduce the child’s defensive stance and open space for dialogue.

#### Clarifying the situation narrative (technique 10)

Teachers frequently supported children in constructing a coherent, sequential account of what had happened, providing organising scaffolding for experiences that felt fragmented or overwhelming.

*(Incident 228)*

Teacher: *Let us go back to where it started. You were doing maths, then [Child B] came in—what happened next?*

The co-construction of a narrative appeared to help children make sense of complex social sequences, creating a basis from which alternative interpretations or responses could be considered.

## Discussion

### Overview of findings

The pattern of findings is theoretically coherent within the mentalization model (Fonagy et al., 2002; Bateman et al., 2023). The most effective techniques – those explicitly focused on exploring the mental states of others and of relational dynamics (Techniques 5 and 6) – engage the most explicitly social and relational dimensions of mentalizing. When a dysregulated child is invited to consider how a peer felt, or what is happening between themselves and the teacher, attention is redirected from the child’s own overwhelmed internal state toward a shared social reality. This may interrupt the psychic equivalence mode – in which internal experience is treated as unquestionable external fact – that characteristically accompanies dysregulation (Bateman et al., 2023). The relatively lesser effectiveness of stabilising techniques (Category 1) is consistent with MBT’s account that containment creates conditions for mentalizing without itself constituting its active ingredient. The failure of Addressing Contradictions and Clarification and Exploration is also theoretically predicted: both techniques require cognitive operations that are unavailable when arousal is highest.

This mixed-methods study examined the use and effectiveness of Mentalization-Based Interventions (MBIs) in an educational context. Across 815 interactions from 259 classroom incidents between teachers and children, the findings demonstrated that MBIs were generally more effective than non-mentalizing approaches in resolving disruptive behaviour and emotional dysregulation.

The coding frame used for analysis was adapted from Muñoz Specht et al. (2016). Inter-rater reliability testing demonstrated strong agreement between coders, supporting the robustness of the framework in this new context. All three mentalization categories outperformed non-mentalizing strategies, addressing the study’s first research aim.

Both incident severity and interaction severity were examined as predictors of resolution. Incidents rated at severity level 3 were significantly less likely to be resolved overall.

Turning to the second research aim, the analysis of specific mentalization techniques revealed that 11 of the 13 examined techniques outperformed the non-mentalizing baseline. The exceptions were Clarification and Exploration and Addressing Contradictions. Qualitative data shed light on these results. Two techniques stood out as particularly impactful: Exploring Mental States of Others and Exploring Mental States in Relationships.

### Relevance to previous research

The findings of this study demonstrate the effectiveness of MBT interventions in resolving classroom incidents, with higher levels of mentalization corresponding to a greater likelihood of resolution. This underscores the central role of teachers’ mentalizing capacities in shaping classroom behaviour as seen with the parallel impact of SEL initiatives (Jennings et al., 2017). The findings are broadly consistent with the direction of Valle et al. (2016) Thought in Mind Project, which showed that students taught by a mentalizing-trained teacher demonstrated gains in higher-order belief understanding relative to controls. However, direct comparison is limited by fundamental design differences: Valle et al. used a quasi-experimental approach with pre- and post-measures of students’ own mentalizing capacity, whereas the present study used naturalistic observation with incident-level analysis of resolution outcomes. The two studies address complementary questions and should be read as mutually supporting rather than directly replicating each other.

These findings raise the theoretical possibility – consistent with attachment theory (Fonagy and Target, 2002) – that sustained teacher use of mentalizing might progressively strengthen children’s own reflective capacity, creating a virtuous cycle of improved mentalizing and reduced dysregulation. However, this remains a hypothesis: the present study was designed to examine incident-level resolution outcomes and cannot provide evidence about developmental change over time. Testing this hypothesis would require longitudinal designs tracking children’s reflective functioning alongside observational data across an academic year. The findings also align with Ensink et al. (2016), who reported that children who mentalize more effectively with attachment figures experience fewer depressive symptoms.

The comparison with the CAPSLE programme (Twemlow et al., 2005) adds nuance. While both this study and CAPSLE point to the effectiveness of mentalization-based approaches in managing misbehaviour, the focus differs. CAPSLE emphasises whole-school strategies to reduce aggression and bullying, whereas the present study examined naturalistic classroom incidents, including episodes of emotional dysregulation. The study found a significant main effect of severity: Level 3 incidents were significantly less likely to be resolved (OR = 0.29), confirming that the most severe incidents present the greatest challenge regardless of technique used. However, no statistically significant interaction between specific technique and severity level was detected, meaning we cannot conclude from the current data that any particular technique was differentially effective for high-severity incidents. Given the small number of Level 3 incidents involving any individual technique, the study was almost certainly underpowered to detect such interactions – this constitutes an important limitation rather than evidence that technique effectiveness does not vary with severity.

The results also align with strategies used in MBT-C, particularly the emphasis on holding a mentalizing stance and adopting a curious, relationally focused approach to children’s challenges (Midgley et al., 2017). The success of *Exploring Mental States in Relationships* in this study parallels the therapeutic use of this technique in MBT-C, while the importance of empathy, support, and validation likewise resonates across both contexts. This mentalizing stance also aligns with trauma-informed teaching practices incorporating the validatory approach of *“what is happening with you?,”* building and sustaining relationships between teachers and children (Thomas et al., 2019).

A divergence was noted in the role of play. In MBT-C, play is a central vehicle for developing awareness of mental states, providing a low-threat space in which children can explore perspectives, emotions, and scenarios without real-world consequence (Fonagy and Target, 1996; Midgley et al., 2017). In the present study, explicitly play-based techniques did not feature in the observed classroom practices beyond occasional playful tones. This absence is theoretically significant: the structured, task-oriented nature of classroom life may not easily accommodate the kind of open-ended play central to MBT-C. Whether certain classroom activities – collaborative tasks, role-play exercises, games with social-dilemma structures – serve analogous mentalizing functions is an interesting question that the present study cannot address but that warrants direct investigation in future research.

The poor performance of *Addressing Contradictions* may reflect the risk of appealing to logic when a child’s capacity to mentalize has temporarily “gone offline” (Bateman et al., 2023). Used in moments of heightened dysregulation, this strategy may feel invalidating or confrontational, as the child is not in a position to engage reflectively. This risk is heightened when affective attunement has not yet been established: without a foundation of felt understanding, contradiction is more likely to intensify dysregulation than to resolve it. The specific risk is that when a child is in psychic equivalence mode – the state in which beliefs feel as certain as external reality – a logical challenge feels not like an invitation to reflect but like a threat to something real. MBT guidance therefore emphasises pairing contradiction with validation and ensuring relational safety before employing it (Bateman et al., 2023). This may be due to deficits in mentalization skills or that an intervention is too demanding or overwhelming. MBT guidance on addressing defiance highlights the value of combining validation with a subtle challenge or “twist” to gently destabilise entrenched thinking (Bateman et al., 2023).

The effectiveness of *Exploring Mental States in Relationships* is consistent with its therapeutic role in MBT-C, where it is used to draw children’s attention to the therapist’s mind (Midgley et al., 2017). In classrooms, teachers appeared able to adapt this technique by prompting children to attend to their perspective, thereby supporting resolution. This may reflect the broader process of social referencing, whereby children calibrate their responses by observing others (Sorce et al., 1985). Similar findings in adults show that calm demonstrators can reduce perceived threat (Golkar et al., 2016), suggesting that children look to teachers for guidance when interpreting challenging situations. However, Bateman et al. (2023) caution that relational mentalizing is best employed when a child is already relatively well-regulated. It is therefore possible that teachers selected this technique when resolution was already underway, contributing to the apparent effectiveness observed in the data. The possibility of reverse causality – that teachers selected relationally focused techniques precisely when resolution was already underway – applies to both Technique 5 and Technique 6. For both, it is possible that teachers identified a window of reduced arousal and chose to use more complex mentalizing techniques in that window, such that the technique codes the fruit of successful de-escalation rather than its cause. Future research should model technique timing within incidents and test whether Techniques 5 and 6 are disproportionately coded in the final interactions before resolution.

This may reflect the broader process of social referencing, whereby children calibrate their responses by observing others (Sorce et al., 1985). While noting that applying this concept to school-aged children interpreting teachers’ responses during conflict situations extends the original paradigm, originally documented in infants, considerably; it is offered as a theoretical analogy rather than a direct empirical claim. Similar findings in adults show that calm demonstrators can reduce perceived threat (Golkar et al., 2016), suggesting that children look to teachers for guidance when interpreting challenging situations.

The complementary success of *Exploring Mental States of Others* may stem from the salience of the peer environment. Research shows that young adolescents are especially sensitive to peer dynamics (Crone and Dahl, 2012), and supportive peer relationships are strong predictors of positive psychosocial outcomes (van Harmelen et al., 2017). By inviting children to consider the experiences and needs of their classmates, teachers effectively harnessed this sensitivity to the peer environment, enabling perspective-taking and facilitating resolution.

A broader theoretical mechanism through which mentalization-informed teaching may produce lasting change – beyond the resolution of individual incidents – is epistemic trust: the willingness to accept communicated knowledge as relevant and applicable to one’s own situation (Fonagy and Allison, 2014; Duschinsky and Foster, 2021). When a teacher adopts a genuine mentalizing stance – marked by curiosity, validation, and openness about their own mental states – they signal to the child that the relationship is trustworthy and that the teacher’s perspective is worth taking in. This may be the mechanism through which individual mentalizing interactions contribute not only to immediate de-escalation but to longer-term shifts in children’s openness to relational learning. Fonagy and Allison (2014) argue that epistemic trust is activated when a communicator demonstrates that they understand the listener’s perspective – precisely what mentalizing techniques are designed to achieve.

### Strengths and limitations

A central strength of this study is its naturalistic design, capturing authentic teacher–child interactions in real classroom contexts. The use of audio recordings, transcripts, and live observational data strengthened accuracy, while the coding scheme – adapted from an established therapeutic framework – demonstrated strong inter-rater reliability, confirmed by high Cohen’s kappa coefficients.

Despite these strengths, limitations remain. The study lacked direct experimental control, and multiple uncontrolled variables may have influenced interactions, meaning causal claims cannot be made about the relationship between specific mentalizing techniques and incident resolution. Because the coding frame was derived from a therapeutic setting, there are inevitable differences in how strategies translate to classroom practice. This raises the likelihood that teachers employed mentalization strategies not captured within the framework, limiting comprehensiveness.

Researcher bias also posed challenges, as interpretations of tone, intent, and non-verbal communication were necessarily subjective. The absence of video recordings meant that non-verbal communications, a key part of the mentalizing process (Bateman et al., 2023), were left to inference, and tonal nuances could easily be misread. A central limitation is the reliance on audio recording as the primary data source. Mentalization is fundamentally a multimodal process: tone of voice, facial expression, gesture, proximity, and gaze all carry significant relational and affective information that audio recording can capture only partially. Non-verbal signals such as the softening of a child’s posture, the re-establishment of eye contact, or the unclenching of hands, may indicate that an intervention is having an effect before any verbal response is produced, and their absence from the data is a meaningful gap. Future studies should incorporate video recording and validated coding schemes for non-verbal communication.

Determining the precise moment of resolution also proved difficult. Teachers often used several techniques sequentially, and resolution may have reflected a cumulative process rather than the final technique coded. A further limitation concerns attribution: in the present study, the technique coded at the moment of resolution receives full statistical credit for the outcome. Incident resolution is likely to be a cumulative process in which earlier techniques create the relational and regulatory conditions under which a final technique succeeds. This attribution structure may systematically inflate the apparent effectiveness of techniques that tend to be used late in incidents and deflate the effectiveness of earlier-deployed techniques such as Clarification and Exploration. Sequential analysis of technique ordering within incidents is needed to address this bias.

The school context substantially limits generalisability. The school maintained a one-to-one teacher-to-student ratio during incidents - a resource-intensive condition unavailable in mainstream settings where average class sizes exceed 26 pupils (Department for Education, 2025). Beyond class size, the staff in this school were working from an explicitly therapeutic, trauma-informed position; mentalizing was integral to their professional identity rather than an add-on to standard teaching practice. This represents a very different educational environment from mainstream schools, where teachers typically receive neither the training nor the relational support structures that characterised this setting. The findings are best understood as demonstrating what is achievable under highly favourable conditions, and future research must examine whether and how the most effective techniques can be adapted for use in contexts where the adult-to-student ratio, staff training, and available time are substantially more constrained. The convenience sampling approach further limits external validity.

### Implications and future research

A key parallel exists between MBT principles and the educational concept of scaffolding (Wood et al., 1976): just as cognitive scaffolding involves building on a learner’s existing capabilities and gradually withdrawing support as competence grows, relational scaffolding involves supporting a child’s capacity to reflect on mental states without overtaking the process on their behalf. MBT explicitly cautions against the therapist doing the mentalizing for the patient, as this creates dependence rather than development (Bateman et al., 2023). In an educational context, this translates to teachers who hold a curious, not-knowing stance that invites children to develop their own mentalizing capacity rather than receiving interpretations ready-made. If this relational scaffolding is sustained across an academic year, it may progressively build children’s reflective functioning in ways that extend beyond behaviour management into broader academic and social learning.

Future research should further investigate the overlap between MBT interventions and classroom strategies. Randomised controlled trials (RCTs) would be especially valuable in testing the causal impact of specific mentalization techniques, although identifying direct causal links is inherently challenging in complex classroom environments. RCT designs face particular challenges in educational settings: individual-level randomisation risks contamination between conditions within the same school; classroom-level randomisation requires large numbers of classrooms to achieve adequate power; and ethical concerns arise around the withholding of a potentially beneficial intervention from control-group children, particularly in vulnerable populations. Cluster randomised designs at the school level with active control conditions represent the most feasible route to causal evidence; quasi-experimental pre–post designs with carefully matched comparison schools could also advance the evidence base in the shorter term. Equipping teachers with practical toolkits to implement these techniques appears to be a promising avenue for building mentalizing environments in schools (Bak et al., 2015; Eppler-Wolff et al., 2020). Taken alongside existing work (Chelouche-Dwek and Fonagy, 2025; Twemlow et al., 2005; Valle et al., 2016), this study adds to a growing body of evidence that supports the value of mentalization in education.

## Conclusion

This study found that mentalization techniques derived from MBT were more effective in resolving disruptive behaviour and emotional dysregulation incidents than strategies that did not involve mentalizing. Of note were techniques focusing on exploring the mental states of others and on relationships, both of which showed a strong impact, possibly reflecting adolescents’ heightened sensitivity to peer dynamics and reliance on social referencing. In contrast, *Clarification and Exploration* and *Addressing Contradictions* did not outperform non-mentalizing strategies. Clarification was often used early on in incidents, providing insight but not resolution, while contradictions risked being perceived as confrontational when used in moments of high dysregulation.

Overall, the findings provide encouraging evidence for the value of applying mentalization techniques from MBT in educational settings. They suggest that teacher-led mentalizing strategies can play an important role in supporting resolution, emotional regulation, and relational understanding in the classroom. Further investigation is warranted to deepen understanding of their effectiveness and to support the development of evidence-based approaches in this emerging field. In doing so, the study takes a first empirical step toward an evidence base for mentalization-informed teaching as a theoretically grounded approach to supporting children’s relational and emotional development in educational contexts—one that future longitudinal and experimental research can build into practical guidance for educators and policymakers.

## Data Availability

The raw data supporting the conclusions of this article will be made available by the authors, without undue reservation.

## Impact statement

This study bridges clinical and educational contexts by examining how mentalization-based techniques, originally developed for therapeutic use, are applied by teachers in everyday educational settings. Using a mixed-methods design and naturalistic classroom data, the findings demonstrate that teacher-led mentalizing strategies can support the management of behavioural and emotional challenges, offering a practical framework for extending therapeutic principles into educational practice.

## Appendix

**Table A:** Incident 76 pre-coding.

Incident no.	Date	Time	Cohort	Trigger	Behaviour	Initial response to behaviour	Resolved?	Child’s response 2
76	07-May	11:35	2	Ab returns to his desk and realizes that someone has opened the Tupperware that he has on his desk	Ab cries out "Jk [Teacher]! Dc tried to open this box without saying anything to me"	Jk [Teacher] says "I'm sorry, that's not very kind, is it?. He should've asked." [soft, calm tone]	Yes	Ab smiles and sits down in his seat

This excerpt illustrates an example of a one-interaction incident. Ab, Dc, and Jk refer to anonymised representations of children and teachers.

**Table B:** Incident 76 post-coding.

Incident no.	Date	Time	Cohort	Trigger	Behaviour	Initial response to behaviour	Resolved?	Which 1/2/3 specific		No. of teachers	Teacher *N*	Student *N*	Child’s response 2
76	07-May	11:35	2	Ab returns to his desk and realizes that someone has opened the Tupperware that he has on his desk	Ab cries out "Jk [Teacher]! Dc tried to open this box without saying anything to me"	Jk [Teacher] says "I'm sorry, that's not very kind, is it?. He should've asked." [soft, calm tone]	Yes	2	6	1	1		Ab smiles and sits down in his seat

This excerpt illustrates an example of a one-interaction incident and its codes. The coding which 1/2/3 = 2 and specific = 6 refers to basic mentalizing techniques and exploring mental states in relationships, respectively. Ab, Dc and Jk refer to anonymised representations of children and teachers.
